# Time is of the essence: The importance of considering biological rhythms in an increasingly polluted world

**DOI:** 10.1371/journal.pbio.3002478

**Published:** 2024-01-30

**Authors:** Eli S. J. Thoré, Anne E. Aulsebrook, Jack A. Brand, Rafaela A. Almeida, Tomas Brodin, Michael G. Bertram

**Affiliations:** 1 Department of Wildlife, Fish, and Environmental Studies, Swedish University of Agricultural Sciences, Umeå, Sweden; 2 TRANSfarm—Science, Engineering, & Technology Group, KU Leuven, Lovenjoel, Belgium; 3 Department of Zoology, Stockholm University, Stockholm, Sweden; 4 Department of Ornithology, Max Planck Institute for Biological Intelligence, Seewiesen, Germany; 5 Institute of Zoology, Zoological Society of London, London, United Kingdom; 6 Laboratory of Aquatic Ecology, Evolution, and Conservation, Department of Biology, KU Leuven, Leuven, Belgium; 7 School of Biological Sciences, Monash University, Melbourne, Australia

## Abstract

Biological rhythms have a crucial role in shaping the biology and ecology of organisms. Light pollution is known to disrupt these rhythms, and evidence is emerging that chemical pollutants can cause similar disruption. Conversely, biological rhythms can influence the effects and toxicity of chemicals. Thus, by drawing insights from the extensive study of biological rhythms in biomedical and light pollution research, we can greatly improve our understanding of chemical pollution. This Essay advocates for the integration of biological rhythmicity into chemical pollution research to gain a more comprehensive understanding of how chemical pollutants affect wildlife and ecosystems. Despite historical barriers, recent experimental and technological advancements now facilitate the integration of biological rhythms into ecotoxicology, offering unprecedented, high-resolution data across spatiotemporal scales. Recognizing the importance of biological rhythms will be essential for understanding, predicting, and mitigating the complex ecological repercussions of chemical pollution.

## Abstract 2

Biological rhythms shape the behavior and ecology of organisms but have been broadly ignored in chemical pollution research. This Essay highlights the importance of biological rhythms for understanding, predicting, and mitigating the complex ecological repercussions of chemical pollution.

## Introduction

Chemical pollution is an urgent and escalating global concern [1,2]. The global production of chemicals, coupled with their release into the environment, has increased 50-fold since 1950 and is expected to triple again by 2050 compared with 2010 [3]. Chemical pollutants have the potential to profoundly alter wildlife biology and ecology, disrupt ecosystems, and pose a serious threat to biodiversity [4,5]. Despite this, our understanding of the ecological effects of chemical pollutants remains rudimentary, leaving us far from effectively managing the risks they pose [5]. As ecotoxicologists and regulators work towards solutions, it is becoming increasingly evident that nature’s inherent complexity challenges our ability to assess the full extent of the risks posed by chemical pollution. In this regard, time has a critical role in structuring biological patterns and processes, including how wildlife are affected by, and respond to, environmental change [6,7]. Indeed, biological rhythms represent a fundamental and universal feature of life, yet our understanding of their ecological and evolutionary underpinnings and consequences remains extremely limited [7,8], particularly within the context of chemical pollution (see also [9,10]).

In this Essay, we aim to highlight the urgent need to integrate biological rhythms into chemical pollution research. We first outline what biological rhythms are and provide an overview of their diversity and significance. We then examine why biological rhythms matter for understanding the ecological impacts of chemical pollution and identify the existing barriers to incorporating biological rhythms into chemical pollution research. Finally, we propose novel methods, tools, and resources that can help to overcome these barriers.

## What are biological rhythms?

**Fig 1:**
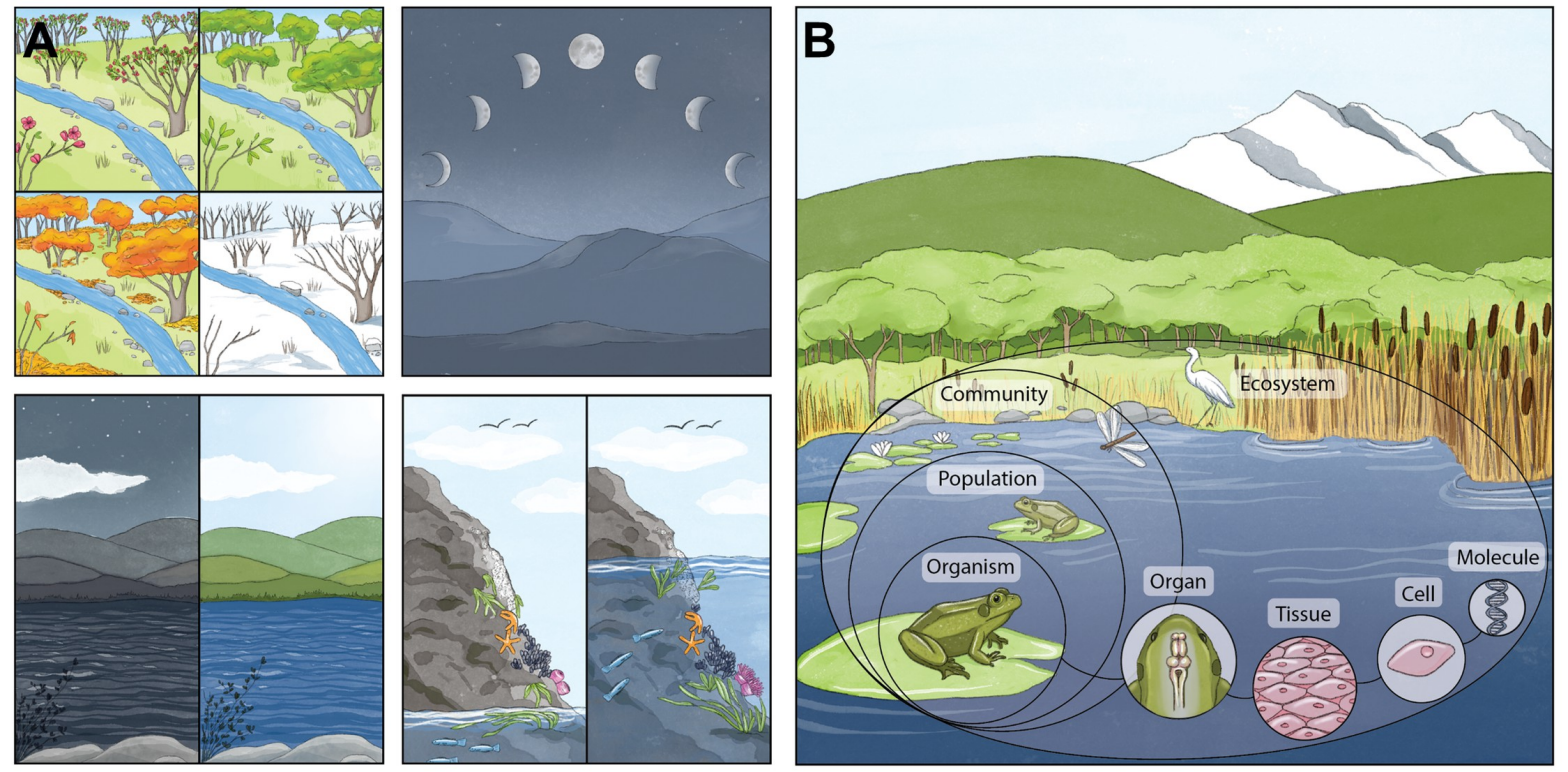
Biological rhythms synchronize the biology of organisms with periodic changes in the abiotic environment. (**A**) Environmental cycles such as seasonality, the lunar cycle, the day–night cycle, and tidal activity are characterized by periodic changes in, among other things, light, temperature, salinity, and oxygen availability. (**B**) Biological rhythms manage the biosphere. They occur across the tree of life and manifest across all levels of biological organization.

Biological rhythms govern the entire biosphere, covering processes as diverse as pulsatile hormone secretion, cyclic variation in appetite and food intake, intertidal activity of coastal and estuarine animals, daily plant leaf movements, the sleep–wake cycle, diurnal vertical migration of animals in oceans and lakes, the menstrual cycle, annual bird migration, and seasonal hibernation. In this Essay, we broadly define biological rhythms as repetitive molecular, physiological, and behavioral processes that occur in anticipation of, or response to, periodic environmental change. Such rhythms are highly diverse in nature (Fig 1), can recur with a frequency that ranges from microseconds to hours (ultradian rhythms), days (diurnal rhythms), or even weeks, months, or years (infradian rhythms), and exist across the tree of life. Some biological rhythms are exclusively regulated by environmental signals, while others are also regulated by internal biological clocks [8,11]. One of the most well-known examples of such an internal regulator is the circadian clock, which operates roughly on a 24-hour cycle and governs the daily rhythms of organisms. While the circadian clock is synchronized or “entrained” by external cues known as zeitgebers (such as light), it runs even in the absence of time-of-day cues (i.e., it has a free-running rhythm [11]). Organisms typically have multiple biological clocks that are spread across several tissues and organs throughout the body, and which control diverse rhythms that are synchronized with each other and with environmental cycles [12]. Furthermore, biological rhythms can be superimposed on one another, giving rise to composite oscillations within biological systems, rather than just following a single cycling frequency [13]. Collectively, biological rhythms coordinate a myriad of essential biological processes and have a fundamental role in regulating life’s processes.

## Why consider biological rhythms in chemical pollution research?

Although the study of biological rhythms (chronobiology) grew from ecological and evolutionary biology roots in the mid-20th century, the field quickly shifted its focus towards the mechanistic underpinnings of biological clocks, and circadian clocks in particular [8]. Thereafter, both fields developed largely independently. Consequently, the majority of insights into the workings and importance of biological rhythms have come from biomedical research. As such, biological rhythms represent a level of complexity that has long been realized but not routinely incorporated into ecology and evolutionary biology [7] and, by extension, global change and chemical pollution research.

**Fig 2:**
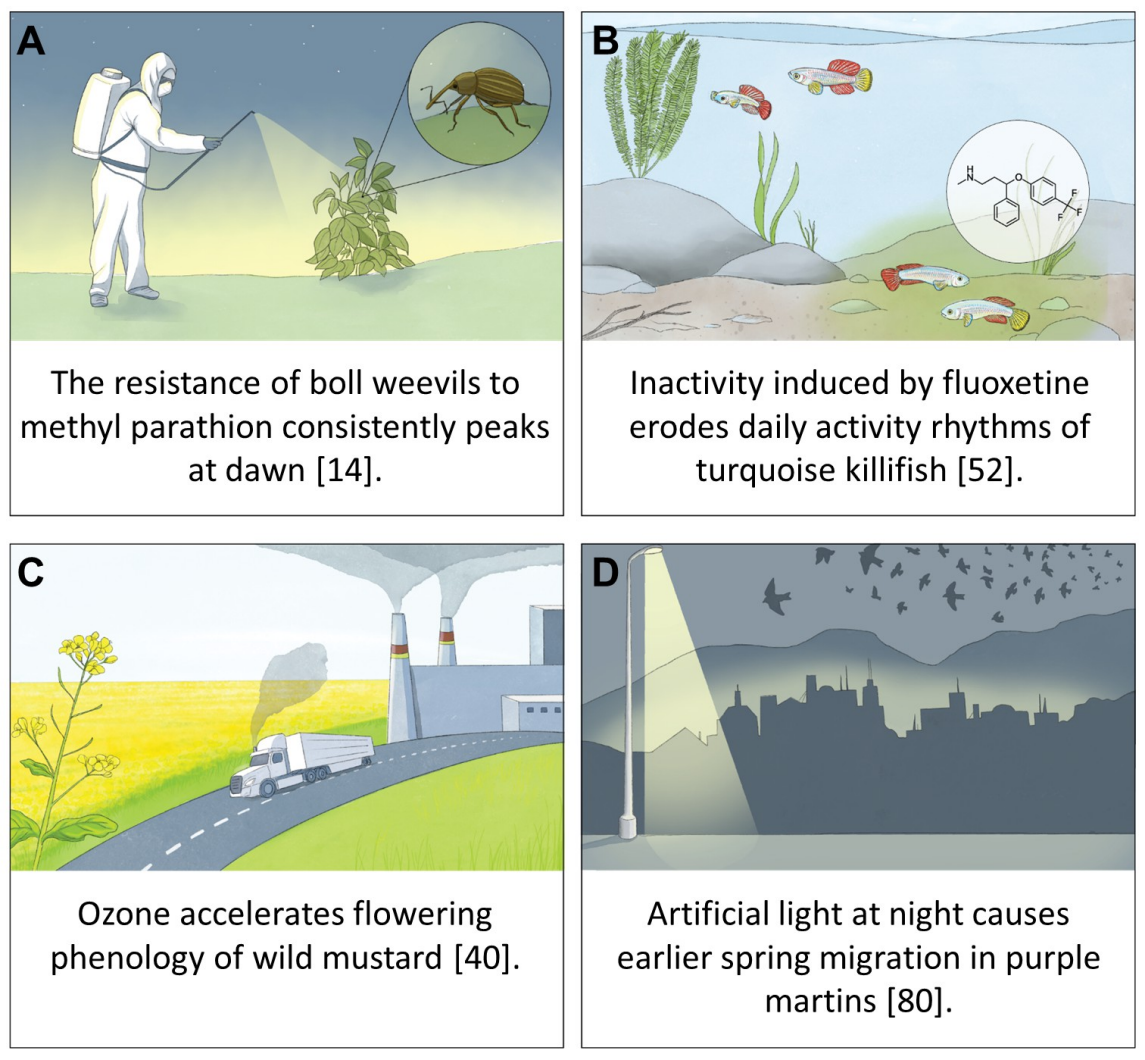
Examples of how pollution can intersect with biological rhythms. Despite having received relatively little research attention to date, the few studies that have tested for potential interactions between chemical pollution exposure and biological rhythms have demonstrated that these factors can interact at various spatiotemporal scales and in diverse biological systems (**A**-**C**). While our focus is on chemical pollution research, chronobiology is clearly relevant to all forms of environmental change (e.g., light pollution, **D**).

Pioneering studies of biological rhythms and chemical toxicity date back to the 1960s. Such studies, which demonstrated circadian variation in pesticide toxicity, already highlighted the importance of considering biological rhythms in ecotoxicology. For example, adult boll weevils (*Anthonomus grandis*) and two-spotted spider mites (*Tetranychus urticae*) exhibit a daily rhythm in their susceptibility to the insecticides methyl parathion and dimethyl 2,2-dichlorovinyl phosphate, respectively. Specifically, weevils consistently experienced the least mortality at dawn [14] (Fig 2), whereas mites were maximally susceptible right after dawn and were least affected just after nightfall [15]. Furthermore, the sensitivity of house crickets (*Gryllus domesticus*) to narcotics (ethyl ether, chloroform, and carbon tetrachloride) peaks during the first part of the nighttime period, corresponding to the species’ peak activity [16]. Conversely, house flies (*Musca domestica*) show maximum sensitivity to the pesticide trichlorfon at dawn and are least sensitive during dusk [17].

While these are not the only studies demonstrating that wildlife susceptibility to toxicants can fluctuate during the day, sometimes by orders of magnitude [18], similar studies remain relatively rare (see also [9,18,19]), and studies that incorporate ultradian or infradian rhythms are even scarcer. This is a cause for concern because failure to integrate biological rhythms into chemical pollution research severely limits our understanding of the ecological and evolutionary responses to, and consequences of, this pervasive form of global change. We contend that biological rhythms should be integrated into chemical pollution research for 3 interconnected reasons: biological rhythms are adaptive (misaligned rhythms can dysregulate biological and ecological functioning); chemicals can affect biological rhythms, and these rhythms can influence the impacts of chemicals; and biological rhythms introduce variation that complicates the interpretation of results. In the following sections, we examine each of these reasons in turn.

## Biological rhythms are adaptive

Biological rhythms are highly adaptive and are believed to be under strong selection, as they govern a broad range of essential processes for wildlife reproduction and survival [6,8]. Specifically, they have a vital role in coordinating the biology of organisms with periodic changes in the abiotic environment, such as changes in light, temperature, salinity, and oxygen availability related to tidal activity, the day–night cycle, the lunar cycle, and seasonality (Fig 1). Further, biological rhythms can coordinate interactions among organisms, including potential mates, competitors, parasites, prey, predators, and symbionts (e.g., the microbiome). For example, dominant brown trout (*Salmo trutta*) forage most intensely from dusk until dawn to maximize food intake and minimize predation risk, whereas the foraging activity of subdominant trout peaks at alternative times of the day to minimize competition [20]. Such rhythms are not necessarily rigidly structured but can shift to help organisms respond to changes in their environment [21]. In the previous example, when competition for resources increases, differences in the timing of foraging activity between dominant and subdominant trout become more pronounced [20]. Similarly, Norway rats (*Rattus norvegicus*) that are generally nocturnal can shift to daytime foraging to avoid predation by nocturnal red foxes (*Vulpes vulpes*) [22]. In some cases, organisms can shift to or from arrhythmicity altogether, such as when male sandpipers (*Calidris melanotos*) shift from daily rhythmicity to almost round-the-clock activity during the breeding season to increase their reproductive success [23].

Although organisms can be equipped to cope with changes in the environment through plastic shifts in their rhythms, some may be far less able to instantly cope with abrupt and/or unpredictable changes than others [8]. In such cases, the timing of biological rhythms may become mismatched with environmental cues, as is the case when we experience jet lag: Travel across time zones shifts our circadian clock out of alignment with local time, which can lead to fatigue and reduced performance [24]. Jet lag in humans is temporary, as the circadian clock eventually resynchronizes, but prolonged misalignment of biological rhythms can result in substantial fitness consequences. For example, misalignment of the circadian rhythm with the 24-hour day–night cycle can come at a physiological cost that accelerates the aging process and decreases life span in rodents and primates [25]. In *Drosophila* fruit flies, experimentally induced desynchronization between rhythmic gene expression in the fat body (a mass of tissue used for energy storage) compared with the brain results in a lower reproductive output [12]. In fact, dysregulation of biological rhythms can often directly lead to various pathologies [26]. Shift work, altered timing of sleep on work days compared with non-work days (or “social jetlag”), and exposure to light-at-night can lead to dysregulations in humans that have been associated with metabolic disorders such as diabetes [27], cardiovascular diseases [28], cancer [29], and various mood disorders [30]. Similar effects occur in experimental animal studies, including the promotion of cardiomyopathy by circadian disruption in hamsters [31], as well as obesity [32] and anxiety-like behavior [33] in mice. Further, there is mounting evidence for a link between circadian clock disruption and increased cancer risk in mice [34]. Naturally, such effects could have strong implications for the survival and reproductive success of wildlife and may have far-reaching population-level impacts.

Beyond direct consequences for the health and performance of organisms, desynchronization of biological rhythms with environmental cycles or with those of other organisms can cascade through different levels of biological organization and have far-reaching ecological repercussions [6,35]. Desynchronized activity rhythms can disrupt social activities such as mating, parental care, or group foraging, which may have substantial demographic consequences [36], and novel “timescapes”(i.e., time periods with fitness-relevant heterogeneity in (a)biotic factors) could fundamentally alter species interactions when species perceive and respond differently to timescape changes [6,37]. This could lead to desynchronization along the food web in, for example, seasonal activity timing [38], and ultimately affect the functioning of whole ecosystems [39]. For example, exposure of wild mustard plants to ozone reduces vegetative growth but accelerates their flowering, which can foster desynchronization between plant and pollinator activities (Fig 2) [40]. Conversely, symbiont–host interactions can contribute to the stabilization and coordination of circadian rhythms within the host organism. Specifically, the microbiome may be able to moderate the host’s response to external environmental cues, ensuring that internal biological processes remain synchronized with the external day–night cycle. This, in turn, promotes overall circadian synchrony within the host, thereby buffering against rapid environmental fluctuations [41].

## Chemicals can affect biological rhythms and vice versa

**Fig 3:**
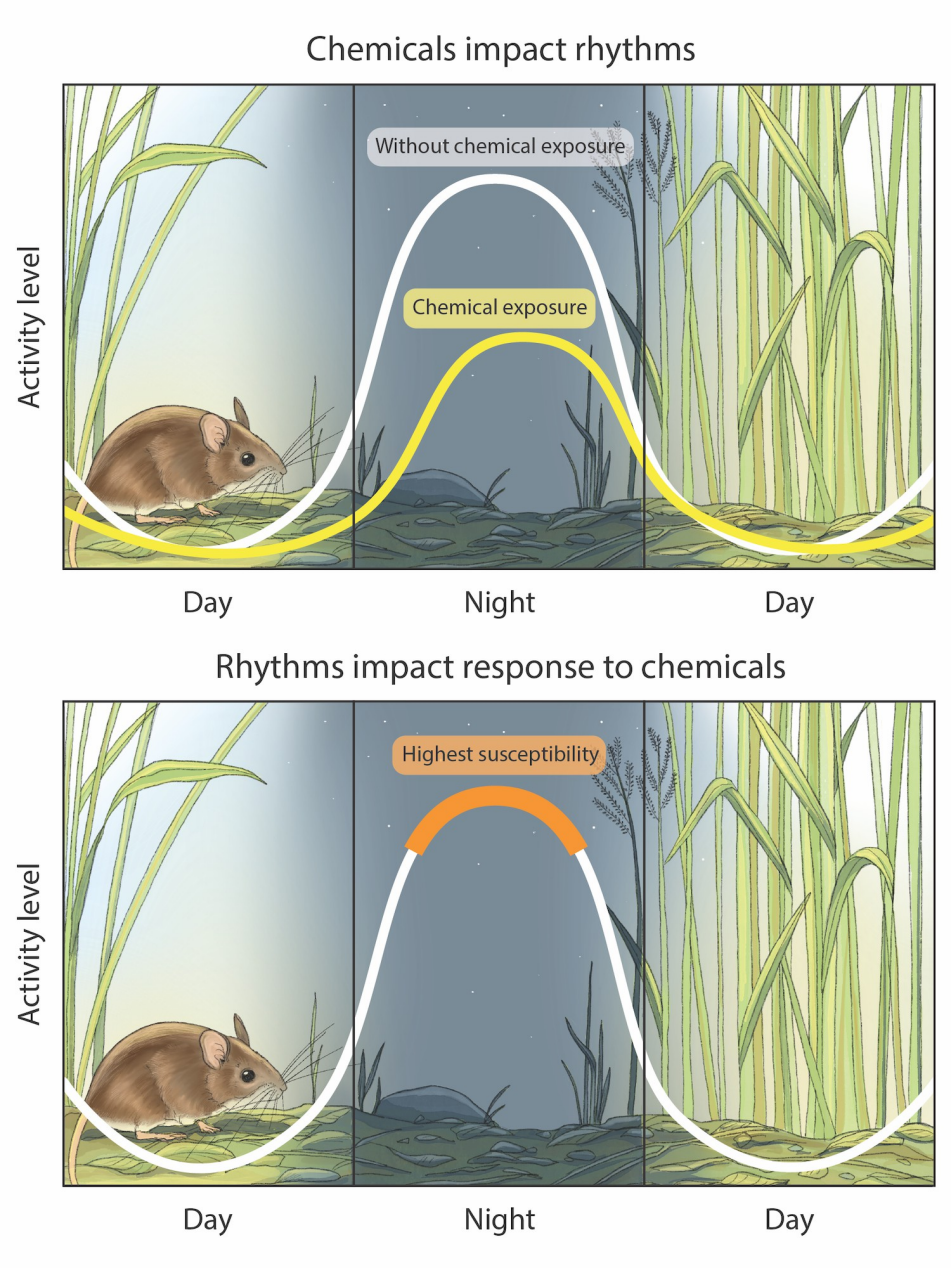
Illustration of the reciprocal relationship between chemical pollutants and biological rhythms. Chemical pollutants can disrupt biological rhythms, and, conversely, biological rhythms can influence an organism’s susceptibility and/or response to chemicals. The example presented here is one of many possible scenarios and is used for illustrative purposes.

It is increasingly evident from the biomedical literature that many emerging forms of pollution, including (inappropriate) exposure to biologically active chemicals (e.g., pharmaceutical drugs or endocrine-disrupting chemicals) can profoundly disrupt biological rhythms. Indeed, recent biomedical research has begun to investigate the direct interactions between disruptions in the human circadian system, mood regulation, and drug use. Even though our understanding of these complex relationships is still in its early stages, there is mounting evidence that several pharmaceutical compounds can modulate circadian rhythms in both humans and animal model species [42] and that inappropriate use of drugs may even disrupt the circadian system altogether [43] (Fig 3). In humans, disruptions to the circadian system have furthermore been linked to a higher usage of hypnotic medication [44] and can increase the risk of drug dependence and addiction [45]. Vice versa, circadian rhythms can affect the pharmacokinetics and pharmacodynamics—including the biological effects—of drugs, to the extent that there is ongoing research into the potential for chronotherapy to improve the efficacy and safety of pharmaceutical agents [42]. The absorption, distribution, metabolism, and excretion of drugs and other compounds are influenced by circadian rhythms [46], such that their effects may vary over the course of a day (Fig 3).

Similar interactive effects between rhythmicity and the effects of chemical pollutants on wildlife are likely to be far more widespread and important than is currently appreciated. At the moment, over 350,000 chemicals are registered for commercial use worldwide [47], of which hundreds are routinely detected in all studied environmental compartments [4,48]. Many of these chemicals may not be present at high enough environmental concentrations to result in direct mortality of wildlife but can nevertheless trigger biological changes, even at extremely low concentrations, that compromise the health of our ecosystems (e.g., per- and polyfluoroalkyl substances (PFAS), pharmaceutical agents, polychlorinated biphenyls (PCBs)) [1,48]. In fact, when the International Union for Conservation of Nature compiled the Red List in 2022, more than 11,500 out of 83,669 assessed animal species were considered to be impacted by chemical pollution [4,49]. While our current knowledge about chemicals interfering with the regulation of circadian rhythms (so-called “circadian disruptors”) is extremely limited, at least 40 chemical pollutants were recently identified that interfere with circadian rhythms in fish at environmentally realistic concentrations [10]. These included steroid hormones, metals, pesticides and biocides, PCBs, neuroactive drugs, and cyanobacterial toxins (which are becoming more widespread due to nutrient pollution and climate warming). For example, neuroactive drugs such as selective serotonin reuptake inhibitors (SSRIs) are commonly used to treat depression or other mental illnesses but are also known to interfere with human circadian rhythms and the sleep–wake cycle [30,42]. Serotonin is also involved in the regulation of circadian rhythmicity and associated behaviors in fish [50], such that exposure to SSRIs has the potential to disrupt circadian rhythms in wildlife [51]. Indeed, chronic exposure to an environmentally relevant concentration (28 ng/L) of the antidepressant fluoxetine entirely eroded daily activity patterns in *Nothobranchius* killifish, suggesting strong circadian disruption [52] (Fig 2). This finding was recently replicated in *Gambusia* mosquitofish after as little as 3 days of exposure to 30 to 300 ng/L of fluoxetine [53] and is consistent with the earlier finding that 96 hours of exposure to a mixture of organic contaminants, including fluoxetine, eroded daily activity patterns in male mosquitofish [54].

Given that our current knowledge about potential circadian-disrupting chemicals is extremely limited [10], it is likely that many more chemicals exist that can interfere with circadian rhythms in wildlife, or with biological rhythms more generally. In support of this expectation, over half of the top 100 best-selling drugs in the United States, and over 100 of the World Health Organization’s list of essential medicines, directly target the product of rhythmic genes [55]. The release of such chemicals into the environment is concerning given that disruption of biological rhythms by chemicals can interfere with organismal survival and reproductive success and, in turn, result in far-reaching population-level and ecological consequences. The issue may be exacerbated by the fact that several molecular components of xenobiotic metabolism and detoxification mechanisms, including cytochrome P450s [56] and antioxidant enzymes [57], seem to be, at least in part, regulated by circadian rhythms. This suggests that even chemicals that do not directly interfere with the circadian system could affect organisms differently depending on the timing of administration or observation [10]. However, this is only very rarely taken into account in ecotoxicological research.

## Biological rhythms introduce variation

Even in the absence of potential interactive effects between chemicals and rhythmically controlled systems, it is essential to appropriately control for the possible confounding effects of rhythmicity in ecotoxicological research. For example, many molecular biomarkers [58] or behaviors [52] commonly considered in ecotoxicological experiments show daily variation in their expression. If experimental sampling is not time-controlled, biological rhythmicity may introduce substantial variation (or “noise”) that results in either false positive or false negative results or that could lead to inaccurate conclusions regarding the magnitude of the effects of chemicals. Accommodating the potential effects of sampling time is therefore critical to the reliability and reproducibility of ecotoxicological data. Furthermore, it is important for researchers to consider that not only light but also many other factors can be important zeitgebers that influence biological rhythmicity. These include temperature, social interactions, and food availability [7]. For example, nocturnal mice can shift their activity patterns to become diurnal when exposed to cold or hunger [59]. In *Drosophila* fruit flies, restricting food intake to a specific time of day during which feeding is typically low can desynchronize internal rhythms. This desynchronization does not directly affect food intake but instead leads to lower reproductive output [12]. Not accounting for such effects can introduce significant variation within and among studies and undermine test–retest reliability in chemical pollution research.

## Barriers and solutions to incorporating biological rhythms into ecotoxicology

The lack of research investigating the interactions between chronobiology and chemical pollution is perhaps not surprising, considering the intensive sampling that is often required to measure biological rhythms [60]. For example, such analysis often requires repeated animal handling for assays (e.g., behavior, metabolic rate, and blood samples) over multiple time points, presenting logistical, economic, and even ethical constraints for certain species. Some of these concerns may be further exacerbated when considering longer-term biological rhythms, where animals need to be tracked across several months (e.g., infradian rhythms such as seasonal changes, migration, or hibernation). What is more, some aspects of biology are difficult to assess continuously and noninvasively altogether. For example, the measurement of (rhythmic) gene expression in animals can be hampered by the invasiveness of physical sampling of secretions or tissue (or sometimes the entire animal) after capture [58,61]. The expense of some techniques can also constrain the number of samples that researchers can analyze. These challenges can substantially limit the feasibility of studying the effects of chemical pollution on biological rhythms (and vice versa).

Fortunately, recent technological advances can help to overcome many of these issues. Improved sensitivity of noninvasive hormonal assays now allows repeated sampling of physiological traits without extensive animal handling, as seen with the increasing use of water and feces samples to measure cortisol or corticosterone expression in organisms including fish [62], aquatic and terrestrial dwelling amphibians [63], reptiles [64], birds [65], and mammals [66]. By sampling structures such as feathers, researchers can also gain insight into the physiological history of an animal, including feather growth rates, hormonal levels, and exposure to pollutants [67,68]. Developments in laboratory hardware and software have also considerably improved the ease of repeatedly recording behavioral observations over multiple time points (reviewed in [69]). In particular, high-resolution infrared-sensitive cameras are increasingly being used to continuously record the behavior of animals under both light and dark conditions, across multiple days. For example, infrared-sensitive cameras have been used to record how the insecticide endosulfan influences circadian rhythms in several parasitoid wasp species (*Leptophilina* spp.) [70]. Furthermore, the increasing availability of high-throughput, often open-source, automated animal tracking technologies has reduced many of the economic and logistical constraints encountered when continuously recording the behavior of animals (see [69,71] for reviews). In combination with infrared-sensitive cameras, these automated tracking technologies allow the recording and measurement of animal behavior in the laboratory for extended periods, as was recently shown in research investigating the behavioral response of eastern mosquitofish (*Gambusia holbrooki*) to the pharmaceutical pollutant fluoxetine [53]. Technologies that measure animal behavior through disruptions in electric fields have also successfully been used to record the effects of chemical pollutants on biological rhythms in fish [54,72].

Improvements in both the cost and utility of field-based technologies are also enabling the measurement of biological rhythms in wild animals under seminatural and natural conditions at an unprecedented resolution and scale. In particular, advances in remote-sensing and biologging provide access to an enormous amount of high-resolution data capable of tracking both short- and long-term biological rhythms in the field [73–75]. For example, high-resolution three-dimensional tracking via acoustic telemetry allowed the measurement of daily activity rhythms in wild Arctic char (*Salvelinus alpinus*) over a full annual cycle [76]. These techniques can be implemented at large spatial and temporal scales, allowing the measurement of long-term biological rhythms (e.g., seasonal variation or migration) that cannot be quantified easily, or at all, in the laboratory. Similarly, biologging devices are increasingly available to record biological rhythms in suborganismal physiological traits in wild animals, as was recently shown in the measurement of brain activity and heart rate during sleep cycles in wild northern elephant seals (*Mirounga angustirostris*) [77]. Although these effects have been largely unexplored in chemical pollution research, key insights can be gained from the light pollution literature, in which researchers have taken advantage of remote-sensing technologies to investigate the influence of artificial light on the timing and characteristics of long-range migratory patterns [78–80] (Fig 2). The implementation of such remote-sensing technologies in combination with slow-release chemical implants [81,82] will provide important information on the role of chemical pollutants in mediating biological rhythms in populations under ecologically realistic conditions.

Although our primary focus has been on chemical pollution, it is important to emphasize that chronobiology is also relevant to other forms of pollution and environmental change. Factors such as light, noise, and temperature can impact biological rhythms [83–85], and, conversely, biological rhythms can influence the effects of these factors on other aspects of biology. A better understanding of various forms of environmental disturbance, including those beyond chemical pollutants, will therefore require the integration of chronobiology with ecological and evolutionary frameworks [7]. Nevertheless, the relevance of chronobiology is better recognized in some fields of environmental pollution research than others. In light pollution research, for example, biological rhythms are deemed important, likely because light is a well-established, critical zeitgeber that regulates biological clocks [86]. As a result, there are many examples of light pollution studies that directly measure effects on biological rhythms or at least consider biological rhythms when designing sampling protocols [87–89]. Such studies, together with other biomedical and chronobiological research, offer useful resources for ecologists who are seeking to integrate chronobiology into their own research.

## Conclusions

Biological rhythms occur across the tree of life and manifest across all levels of biological organization. Recognizing their fundamental role in shaping the biology and ecology of species, we contend that integrating biological rhythmicity into chemical pollution research is necessary. Furthermore, incorporating biological rhythmicity into ecological and evolutionary frameworks more broadly will be crucial for fully understanding, predicting, and mitigating the ecological repercussions of chemical pollution. This is particularly significant given that chemicals can disrupt biological rhythms, which, vice versa, govern the efficacy and toxicity of chemicals. In addition, an improved understanding of chronobiology—including ultradian, diurnal, and infradian rhythms, and their combinations—will be essential for aligning data collection with biological rhythmicity and enabling accurate inference regarding the direction and magnitude of effects in chemical pollution studies.

Looking to the future, recent advancements in remote-sensing and biologging technologies are offering unprecedented access to high-resolution data across different spatiotemporal scales, both in laboratory settings and in (semi)natural field conditions, which will facilitate the integration of biological rhythms into research efforts. The field can also advance by drawing upon insights from the biomedical and light pollution literature, where the underlying mechanisms and functional significance of biological rhythms have been more extensively studied, and which can greatly benefit chemical pollution researchers. Recognizing that biological timing is of the essence, we anticipate a promising future for this field as we strive to enhance our understanding and mitigation strategies in an increasingly polluted world.

## Abbreviations

PCB: polychlorinated biphenyl
PFAS: polyfluoroalkyl substance
SSRI: selective serotonin reuptake inhibitor
